# FAMILY HISTORY OF ALZHEIMER’S DISEASE AND LOCUS COERULEUS ACTIVATION IN HEALTHY OLDER AND YOUNGER ADULTS

**DOI:** 10.1093/geroni/igae098.4189

**Published:** 2024-12-31

**Authors:** Elayna Seago, Ya-Yun Chen, Tae-Ho Lee, Ben Katz

**Affiliations:** Virginia Tech, Blacksburg, Virginia, United States; Virginia Tech, Blacksburg, Virginia, United States; Virginia Tech, Blacksburg, Virginia, United States; Virginia Tech, Blacksburg, Virginia, United States

## Abstract

The locus coeruleus (LC) is a small nucleus in the brainstem that produces the majority of norepinephrine in the brain. It engages in reciprocal communication with the frontoparietal network and the salience network, both networks that are critical for attention and undergo significant structural and functional changes during aging. Additionally, the LC is suggested as the primary region of early Alzheimer’s Disease (AD) pathology and its dysfunction may contribute to AD symptomology. It is largely unknown whether family history of AD is linked to LC activity or moderates the association between age and LC functional activity. In this study, functional MRI scans were collected while participants completed the Attentional Network Task. Neural activity in the LC was compared in congruent and incongruent conditions. Twenty-three out of 77 participants had a family history of AD. Participants age ranged from 18 to 74. Multiple regression analysis, controlling for gender, showed no main effect of age on left LC activation (p = 0.289); critically, there was a marginal main effect of family history of AD on left LC activation (p =.088) such that the activation in incongruent compared to congruent condition was higher in participants with a family history of AD than in participants without a family history of AD. We did not find an interaction between family history of AD and age (p =.180) or any right LC effects. These results demonstrate that AD family history is related to left LC activity in both younger and older adults.

